# Effects of fermented herbal tea residue on meat quality, rumen fermentation parameters and microbes of black goats

**DOI:** 10.1186/s13568-023-01610-2

**Published:** 2023-10-03

**Authors:** Mingyue Wang, Longfei Wu, Yongqing Guo, Jiajie Sun, Ming Deng, Guangbin Liu, Yaokun Li, Baoli Sun

**Affiliations:** College of Animal Science, South China Agricultural University, Guangzhou, 510642 China

**Keywords:** Herbal tea residue, Meat quality, Rumen microbiota, Chuanzhong black goats

## Abstract

Herbal tea residue (HTR) is generally considered to be a reusable resource which has still retains considerable proportion of nutrients and active substances. This study aimed to investigate the effects of substitution of whole corn silage with fermented herbal tea residue (FHTR) on meat quality, serum indices, rumen fermentation, and microbes in Chuanzhong black goats. Twenty-two female Chuanzhong black goats (4 months old) with similar weight (9.55 ± 0.95 kg) were selected and randomly divided into two groups. FHTR was used to replace 0% (CON group) and 30% (FHTR group) of whole corn silage in the diets and fed as a total mixed ration (TMR) for Chuanzhong black goats. The adaptation feeding period was 7 days, and the experimental period was 35 days. Results illustrated that the FHTR group had higher value of a* and concentrations of DM and CP and lower rate of water loss (P < 0.05) than the CON group. For the serum indices, goats fed with 30% FHTR had higher (P < 0.05) concentration of CR on day 35. For rumen fermentation, the pH and ratio of acetic acid/propionic acid (AA/PA) in the FHTR group were significantly lower than those in the CON group (P < 0.05). In addition, we studied the goats’s rumen microbial community composition and found that the dominant phyla were *Firmicutes*, *Bacteroidetes*,and *Tenericutes*; and the dominant genera were *Quinella*, *Candidatus_Saccharimonas*, and *Saccharofermentans*. There was a significant difference in the beta diversity of the rumen microbiota between groups (P < 0.05). To sum up, the addition of FHTR can affect the meat quality, serum indices, improved rumen fermentation by adjusted the diversity and function of the rumen microbiota.

**Supplementary Information:**

The online version contains supplementary material available at 10.1186/s13568-023-01610-2.

## Introduction

Herbal medicine tea, also known as Guang-style HT, is composed of seven traditional herbs (jelly grass, frangipani, honeysuckle, chrysanthemum, prunella spike, cloth residue leaves and licorice). These traditional Chinese HT contains a variety of biologically active compounds, such as flavonoids, polysaccharides, and alkaloids (Zhao et al. 2013). Currently, drinking HT is widely because of their has a long history of being used to prevent and treat diseases (Liu et al. 2013) such as essential functions in humans and animals, including treatment for colds (Melnyk et al. 2021); anti-oxidant, anti-inflammatory, anti-proliferative, anti-mutagenic, anti-bacterial, and anti-viral properties (Patel 2022) and cardiovascular diseases (Fu et al. 2018). Researchers find that adding HT to food increased the average lifespan of fruit flies by more than 50% (Shen et al. 2020). However, the HT by-products (herbal tea residues, HTR) have not been effectively used. Conventional incineration, landfill, composting, and other treatments have led to severe environmental pollution (Zhang et al. 2020). Processing and utilization of HTR is of great significance for industrial development and environmental protection. Tea residues (TR) can be used not only as organic fertilizers (Li et al. 2021a) but also as an adsorbent to remove various heavy metal ions (Zhu et al. 2022). HTR is difficult to preserve and digest due to its high fiber and water content. Microbial fermentation can refine the nutrition and preservation time of HTR (Xie et al. 2020). A previous study suggested that, fermented HTR (FHTR) can improve the performance of fattening cattle under heat stress (Zhuang et al. 2021). This potentially indicates that FHTR have the function of improving animal performance.

Mutton is popular among consumers because of its high-quality protein and mineral elements (such as iron and zinc), and amount consumed continues to increase (Wang et al. 2021a), people have higher requirements for the quality of meat products. To date, some plant by-products with many biologically vigorous substances have attracted people’s attention (Chen et al. 2019; Cui et al. 2019). For instance, the addition of flavonoid-rich ginkgo biloba residue meal enhanced the meat quality in Haimen white goats (Chen et al. 2021). The supplementation of ramie to the diet of Liuyang black goats improved the meat amino acid content (Tang et al. 2021). In addition, adding purple corn anthocyanins to the diet can ameliorate the quality of lamb meat by regulating the abundance of several flavor-related genes in the muscle (Tian et al. 2021). Herbal tea residue (HTR) has still retains considerable proportion of nutrients and active substances. Therefore the potential feeding value of the FHTR prompted us to develop a suitable technology for animal production.

To date, few scholars have emphasized whether FHTR can improve meat quality and regulate rumen fermentation and microbial functions after replacing conventional feed ingredients in goats. In the present study, we aim to investigate the effects of substitution of whole corn silage with FHTR on meat quality, serum indices, rumen fermentation, and ruminal bacterial diversity in Chuanzhong black goats. This work provides a basis for using FHTR as a functional feed source for ruminants.

## Materials and methods

### Preparation of FHTR

HTR was provided by Wong Lo Kat Co. LTD (Guangzhou, China), cut into 2–3 cm pieces, and mixed with oat hay (fresh weight 640:360). HTR was added with 2% molasses, and 1% *Lactobacillus plantarum* (Heyuan Jilongxiang Biotechnology Co., Ltd., 5 × 10^9^ colony-forming unit/g) and mixed evenly using a feed mixer. The mixture was pressed into 50 kg per bag for anaerobic fermentation for 20 days. The nutrient compositions of FHTR are shown in Additional file 1: Table S1.

### Experimental design and animal management

This study was conducted in a commercial farm in Zhaoqing City (Guangdong Province, China). Twenty-two female Chuanzhong black goats (4 months old) with similar weight (9.55 ± 0.95 kg) were assigned randomly into two groups. The experimental feed was composed of two levels of FHTR (0% and 30%) as a substitute for WCS (labeled CON and L30, respectively). All of the goats were fed a total mixed ration (TMR). The TMRs were formulated based on the Chinese feeding Standards (China Standard NY/T816-2004). Dry matter (DM), crude protein (CP), and ether extract (EE) were measured following the procedures of the Association of Official Analytical Chemists (AOAC) (2000). Acid detergent fiber (ADF) and neutral detergent fiber (NDF) were examined using the method described in a previous study (Van Soest et al. 1991). Net energy for maintenance (NEM) and net energy for weight gain (NEG) were calculated according to the method provided by the Chinese Feeding Standards (China Standard NY/T816-2004). The dietary ingredients and the nutrient compositions for the trial were shown in Additional file 1: Table S2. The goats were fed twice daily at 8:00 and 15:00, and water was freely available throughout the experimental period. The adaptation feeding period was 7 days, and the experimental period was 35 days. All animal procedures were approved by the Animal Care Committee at South China Agricultural University.

### Measurements and sampling

#### Serum indices

On day 0, 17, and 35, blood samples were gathered from each group via the jugular vein before the morning feeding. The blood samples were placed on the ice for 6 h and then centrifuged at 3000 rpm for 15 min. The serum was placed in a 2 mL sterile EP tube and stored at − 80 °C for further analysis of serum indices. Serum indices, including alanine aminotransferase (ALT), uric acid (UA), creatinine (CR), glucose (GLU), creatine kinase (CK), and lactate dehydrogenase (LDH), were detected using a biochemical auto-analyzer (Hitachi automatic biochemical analyzer 7080, Tokyo, Japan).

#### Meat quality-related indices

Feeding was stopped the day before the end of the experiment. On the last day of the experiment, eight goats from each treatment group were selected randomly and slaughtered according to the method provided by the operating procedures of livestock and poultry slaughtering-Sheep and goat (China Standard NY/T3469-2019). The longissimus dorsi (LD) samples were removed from each carcass at the 11th–13th rib position and divided into two parts. The pH value, meat color, water loss rate, and shear force were tested to evaluate meat quality. All measurements were carried out on the LD samples. The pH of muscles was measured at 45 min and 24 h after slaughter, and the samples were preserved in a refrigerator at 4 °C after each measurement.

Meat color: meat color [lightness (L*), redness (a*), and yellowness(b*)] was measured with a color difference meter (NR10QC, 3NH Company, Shenzhen, China) 45 min after slaughter.

Water loss rate: meat samples were prepared. The fascia and fat were removed and the muscle was cut into long strips weighing 3–4 g. The sample was centrifuged at 1500 rpm and 4 °C for 30 min, and weighed. Water loss rate (%) = (meat sample weight after centrifuging/meat sample weight before centrifuging) × 100%.

Shear force: meat samples were cut into 1 cm thick pieces, vacuum-packaged in non-permeable polyethylene bags, and cooked in a water bath at 85 °C for 10 min, resulting in a core temperature of about 75 °C. The shear force (N) was determined by the c-LM muscle tenderness meter (Northeast Agricultural University Engineering College, Heilongjiang, China).

Meat chemical analysis: Meat samples were cut into pieces and dried in a vacuum freeze-drying machine (ScienTZ-18ND, Ningbo Xinzhi Biotechnology Co, Ltd., Ningbo, CHN). Chemical analyses of CP, EE, and DM were carried out following the method of AOAC. Dried muscle tissue (approximately 100 mg) was put in a hydrolysis tube, After this, 15 mL of 6 mol/L hydrochloric acid solution and four drops of phenol were added. Samples were put in a cryogenic chamber and frozen for 5 min, then sealed under nitrogen filled atmosphere stored for over one hour. Samples were then placed at 110 °C for hydrolysis for 22 h. The obtained hydrolysate was filtered into a 50 mL volumetric flask, and mixtures were vortexed. A 0.2 N sodium citrate buffer (pH 2.2) was added to the test tubes to dissolve the residue and homogenized, then 0.22 μm syringe filter (Sartorius, Göttingen, Germany) was used for filtration. The amino acid contents were analyzed by an amino acid analyzer (L-8900, Hitachi, Japan) (Khalid et al. 2022; Liang et al. 2022). The determination principle is to distinguish amino acids according to their different structure, acidity, alkalinity, polarity, and molecular size.

#### Determination of rumen fermentation parameters

Rumen fluid was collected, and the container was thoroughly cleaned with fresh water between sample collections. The rumen fluid samples were filtered with four layers of gauze, which were subjected high-pressure sterilization. The filtered rumen fluid was separated into two 50 mL centrifuge tubes and three 2 mL cryogenic vials. The cryogenic vial was instantly frozen in liquid nitrogen and stored in a freezer at − 80 °C. The pH of the rumen fluid in a 50 mL centrifuge tube was tested using a portable pH meter, and measurement was repeated three times. The rumen fluid in the other 50 mL centrifuge tube was centrifuged for 15 min at 4000 r/min to collect the supernatant, which was divided into three 15 mL centrifuge tubes. One bottle was used for the determination of VFA content. The other tube was stored at − 20 °C for the determination of ammonia nitrogen (NH_3_–N). The remaining tube was stored at − 80 °C as a backup sample. VFAs, including acetic acid (AA), propionic acid (PA), isobutyric acid (IBA), isovaleric acid (IVA), and butyric acid (BA), were analyzed by HPLC, which was equipped with a Shodex RS Pak KC-811 column (Showa Denko K.K., Kawasaki, Japan) and an SPD-20 A detector (Shimadzu, Kyoto, Japan). Measurement was conducted using the following conditions: eluent of 3 mmol/L HClO_4_, running rate of 1.0 mL/min, and column oven temperature of 50 °C. The concentration of NH_3_–N was measured according to the method of Broderick and Kang (1980): NH_3_–N concentrations: NH_3_–N (mg/100 mL) = C×1.4, where C is the concentration of NH_3_–N (mmol/L) calculated by the standard curve and 1.4 is the conversion factor.

#### Analysis of rumen bacterial community

Total genomic DNA of rumen fluid samples was extracted by CTAB method. DNA concentration was determined using the Nanodrop2000/2000c nucleic acid protein detector (Thermo, Waltham, Massachusetts, USA). DNA quality was evaluated using 2% gel electrophoresis. The V1–V9 regions of the bacterial 16S rRNA gene was amplified using primers 27F (5ʹ-AGAGTTTGATCCTGGCTCAG-3ʹ) and 1492R (5ʹ-GNTACCTTGTTACGACTT-3ʹ) (He et al. 2019). PCR amplification was conducted using the TransStart® FastPfu DNA polymerase kit (TransGen Biotech, Beijing, China). After amplification, the quality control and purification of the PCR products were carried out and high-throughput sequencing was performed by the PacBio Sequel platform. The PacBio off-machine data was exported to bam format files, and LimA software was used to distinguish data according to barcode sequence, save the sequences of all samples in bam format, and use CCS (SMRT Linkv7.0) to correct the sequences. The sequence correction parameters were as follows: the circular-consensus sequence (CCS) = 3, the minimum accuracy was 0.99, and sequence length of less than 1340 bp; sequences with length greater than 1640 bp were removed. The read sequence data were compare with the reference database by using the UCHIME (http://www.drive5.com/usearch/manual/uchime_algo.html) algorithm to detect and eliminate chimeric sequences and obtain clean reads for subsequent analysis (Haas et al. 2011; Edgar 2013). Uparse software was used to cluster all clean reads of all samples. By default, the sequences were clustered into operational taxonomic units (OTU) with 97% consistency, and the representative sequences of OTU were selected (Edgar et al. 2011). Species annotation analysis of OTU representative sequences (threshold set at 0.8–1) was performed using Mothur method and SILVA’s SSUrRNA database (https://www.arb-silva.de/) (Quast et al. 2012). The data of each sample were normalized, and subjected to analyses of alpha diversity analysis and beta diversity. Alpha diversity was calculated using Qiime software (Version 1.9.1), and differences between groups were analyzed using R software (Tang et al. 2021) (Version 2.15.3). Unweighted UniFrac distances were computed using the Phyloseq default script to measure beta diversity. Principal component analysis (PCA) was conducted using the ade4 and ggplot2 packages of R software. Function prediction was detected using Tax4Fun, an R package (Version 2.15.3) for the function prediction based on the16s Silva database (Aßhauer et al. 2015).

### Statistical analysis

Excel 365 was used for the preliminary collation and analysis of experimental data measured. The meat quality indices and rumen microorganism data were analyzed as ANOVA procedure using SAS 9.4 (SAS Inst Inc, Cary, NC, USA). The model used was Yij = µ + Τi + εij, where Yij is the dependent variable, µ is the general mean, Τi is the fixed effect of treatment, and εij is the random error.

Serum indices data were analyzed by MIXED procedure using SAS 9.4 to determine whether they were affected by the interaction of diet and feeding time. The CLASS statement was used to define categorical variables; The MODEL statement was used to define serum indices as dependent variables, and diet, feeding time, and interaction of diet and feeding time was independent variables. DDFM = KENWARDROGER was used to estimate the modified degrees of freedom. REPEATED sentences determined the variance and covariance of the repeated measures. Variance type and covariance structure were used in the auto-regressive models [type = AR(1)]. LSMEANS sentence was used to calculate the average. When the interaction was not significant, ANOVA was used to analyze the effect of diet on serum indices. Experimental data were shown in the table by means and standard error of means (SEM). P < 0.05 indicated significant difference, and P < 0.01 indicated extremely significant difference.

## Results

### Carcass characteristics and meat quality

Table 1 shows the effect of dietary treatment on the chemical composition and characteristics of LD. Compared with CON, the FHTR group had higher a* value, concentration of DM, CP, and lower water loss rate (P < 0.05). No effect due to the dietary treatment was found in EE (P > 0.05). FHTR had no effect on the pH45min, pH24h, L* value, b* value, or shear force of goats (P > 0.05). Table 2 shows the effect of dietary FHTR on the amino acid content of LD. We found that feeding FHTR did not affect the amino acid composition of LD of goats (P > 0.05). The amino acid composition and content in each test group were evaluated according to the scoring standard of the Food and Agriculture Organization of the United Nations/World Health Organization (FAO/WHO). The scoring results are shown in Additional file 1: Table S3. The two groups had similar amino acid scores.

**Table 1:** Effects of FHTR on chemical composition and characteristics of longissimus dorsi (LD) of Chuanzhong black goats

Items	Treatment		SEM	*P*-value
	CON	L30		
DM (%)	19.17	23.30	2.16	0.003
CP (%)	81.05	84.23	1.57	0.001
EE (%)	7.82	7.65	0.18	0.549
L*	53.89	53.41	0.59	0.729
a*	15.2	17.06	0.50	0.040
b*	8.63	8.60	0.15	0.934
pH_45min_	5.99	5.90	0.03	0.196
pH_24h_	5.85	5.75	0.03	0.109
Shear force	47.79	47.74	1.23	0.974
Water loss rate (%)	5.61	4.93	0.01	0.019

*CON* 0% fermented herbal tea residue silage, *L30* 30% fermented herbal tea residue silage, *DM* dry matter, *CP* crude protein, *EE* ether extract, *SEM* standard error of means

**Table 2:** Effects of FHTR on amino acid composition and content of LD of Chuanzhong black goats

Items	Treatment		SEM	*P*-value
	CON	L30		
EAA, g/100 g				
Thr	3.55	3.59	0.04	0.624
Val	3.83	3.85	0.05	0.865
Met	2.03	2.01	0.05	0.508
Ile	3.59	3.65	0.05	0.581
Leu	6.43	6.52	0.07	0.541
Phe	3.23	3.34	0.06	0.434
Lys	7.03	7.15	0.08	0.459
NEAA, g/100 g				
Asp	6.95	7.02	0.10	0.744
Ser	2.91	2.94	0.04	0.692
Glu	11.8	11.8	0.16	0.867
Gly	3.50	3.37	0.04	0.103
Ala	4.39	4.45	0.04	0.369
Cys	0.76	0.8	0.05	0.748
Tyr	2.74	2.78	0.08	0.837
His	2.01	2.37	0.12	0.133
Arg	5.09	5.13	0.04	0.706
Pro	3.10	3.03	0.03	0.206
EAA	29.69	30.11	0.35	0.504
NEAA	43.25	43.69	0.45	0.611
DAA	32.61	32.76	0.32	0.759
TAA	72.94	73.80	0.79	0.556
EAA/NEAA, %	68.65	68.92	0.31	0.567
EAA/TAA, %	40.70	40.80	0.11	0.565

*CON* 0% fermented herbal tea residue silage, *L30* 30% fermented herbal tea residue silage, *EAA* essential amino acid, *NEAA* nonessential amino acid, *DAA* delicious amino acid, *TAA* total amino acids, *Thr* threonine, *Val* valine, *Met* methionine, *Ile* isoleucine, *Leu* leucine, *Phe* phenylalanine, *Lys* lysine, *Asp* aspartic acid, *Ser* serine, *Glu* glutamic acid, *Gly* glycine, *Ala* alanine, *Cys* cystine, *Tyr* tyrosine, *His* histidine, *Arg* arginine, *Pro* proline, *SEM* standard error of means

### Serum indices

Serum indices are listed in Additional file 1: Table S4. Our finding suggested that these serum indices were not affected by the interaction of diet and feeding time. For the serum indices, dietary FHTR supplementation did not influence the concentrations of ALT, UA, CK, GLU, and LDH in the experiment period (P > 0.05). On day 35, goats fed with 30% FHTR had higher CR concentration (P < 0.05).

### Rumen fermentation

Table 3, shows that supplementation with FHTR did not alter the concentrations of PA (P = 0.069), IBA (P = 0.414), and IVA (P = 0.235) between groups. In contrast, the concentrations of NH_3_–N (P = 0.001), AA (P = 0.024), BA (P = 0.019), VA (P = 0.040), and total VFA (P = 0.016) were significantly increased in the FHTR group. The ruminal pH value (P = 0.007) and the ratio of AA/PA (P = 0.026) were significantly decreased by the addition of FHTR to the goat diets.

**Table 3:** Effects of FHTR on the ammonia nitrogen (NH_3_–N) and volatile fatty acids (VFA) of rumen samples of Chuanzhong black goats

Items	Treatment		SEM	*P*-value
	CON	L30		
pH	6.62	6.37	0.51	0.007
NH_3_–N (mg/dL)	13.90	17.74	1.97	0.001
VFA (mmol/L)				
Acetic acid	52.36	66.06	3.16	0.024
Propionic acid	12.34	14.06	0.48	0.069
Isobutyric acid	0.89	0.94	0.31	0.414
Butyric acid	6.16	7.84	0.38	0.019
Isovaleric acid	0.95	1.09	0.58	0.235
Valeric acid	0.47	0.58	0.29	0.040
Acetic acid/propionic acid	4.24	4.68	0.10	0.026
T-VFA	73.14	90.57	14.98	0.016

*CON* 0% fermented herbal tea residue silage, *L30* 30% fermented herbal tea residue silage, *pH* pH value, *VFA* volatile fatty acids, *NH*_*3*_*–N* ammonia nitrogen, *AA/PA* acetic acid/propionic acid, *T-VFA* total volatile fatty acids, *SEM* standard error of means

### Sequencing depth and microbiota diversity

**Fig. 1:**
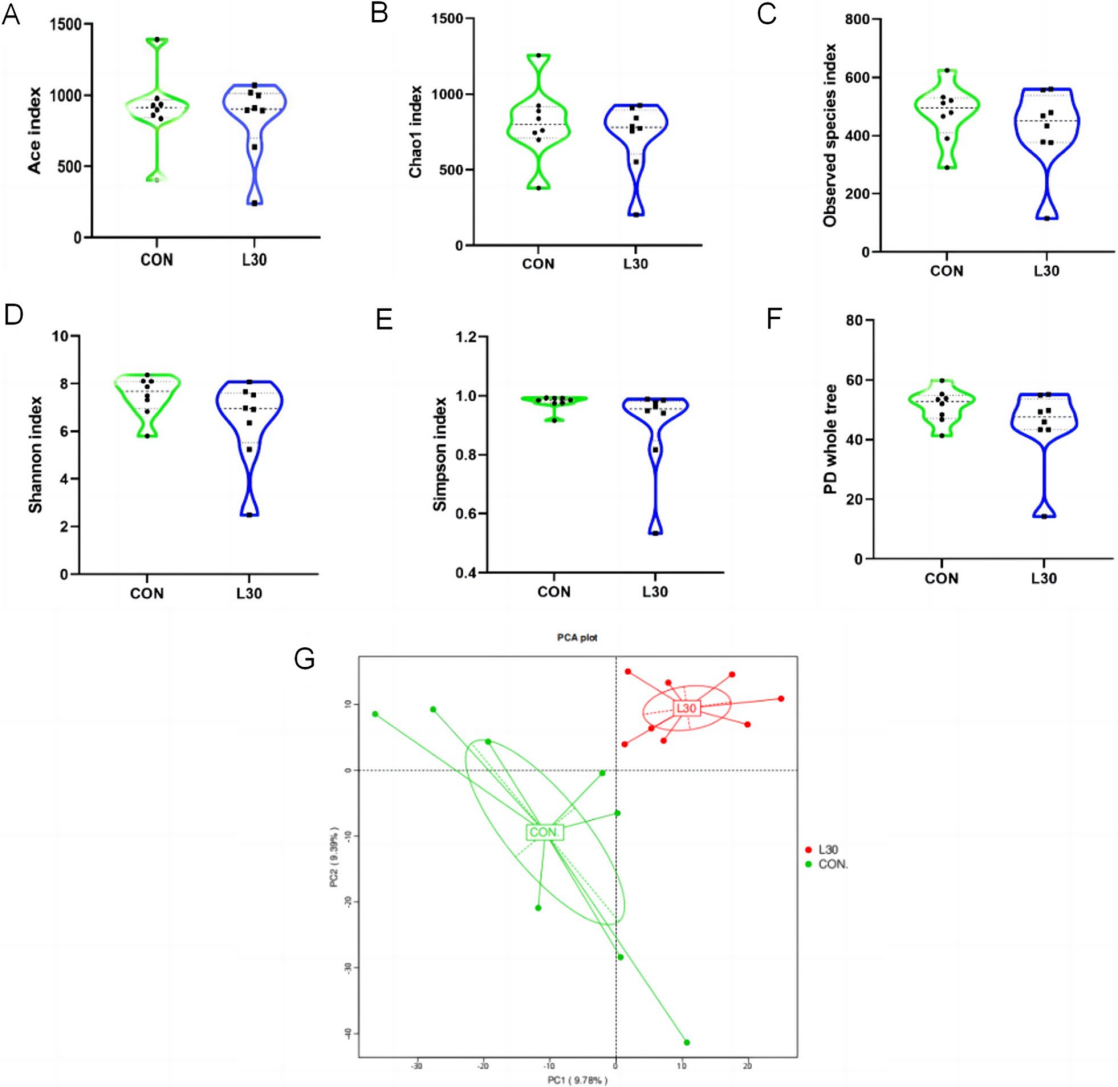
Sequencing depth and fecal microbiota diversity: **A** Ace index; **B** Chao1 index; **C** observed species index; **D** Shannon index; **E** Simpson index; **F** PD whole tree. **G** Principal Component Analysis (PCA)

**Fig. 2:**
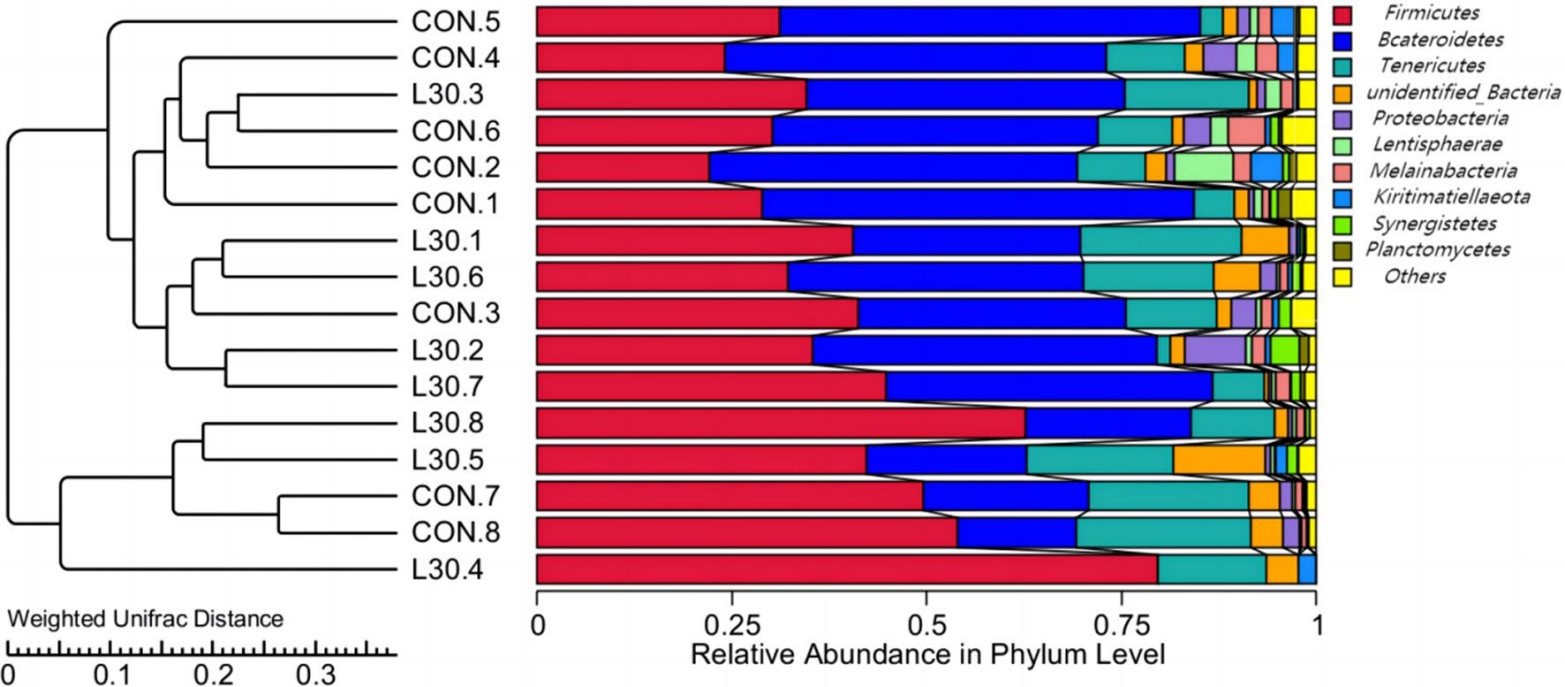
The UPGMA clustering

In our study, the richness indices (Chao1 and Ace), phylogenetic diversity(PD) whole tree, observed species, and diversity indices (Shannon and Simpson) were calculated to reckon the reliability of sequencing and the changes of alpha diversity of sample microbiota (Fig. 1A–F). No significant differences in these indices were found between the groups (P > 0.05). Principal component analysis (PCA) enables the samples to be represented by linear transformation and dimensionality reduction so that the data can be visually presented. As shown in Fig. 1G, the sample distances in each group were relatively dispersed, showing two diverse settlements. The UPGMA clustering results (Fig. 2) were integrated. In addition, analysis of multi-response displacement process (MRPP) analysis similarity (ANOSIM), and permutational multivariate analysis of variance (PERMANOVA) were performed to the rumen microflora in the two treatments (Additional file 1: Table S5), which further proved that there was a significant difference in the beta diversity of the rumen microbiota between groups (P < 0.05).

### Rumen bacterial community composition across different dietary treatments

**Fig. 3:**
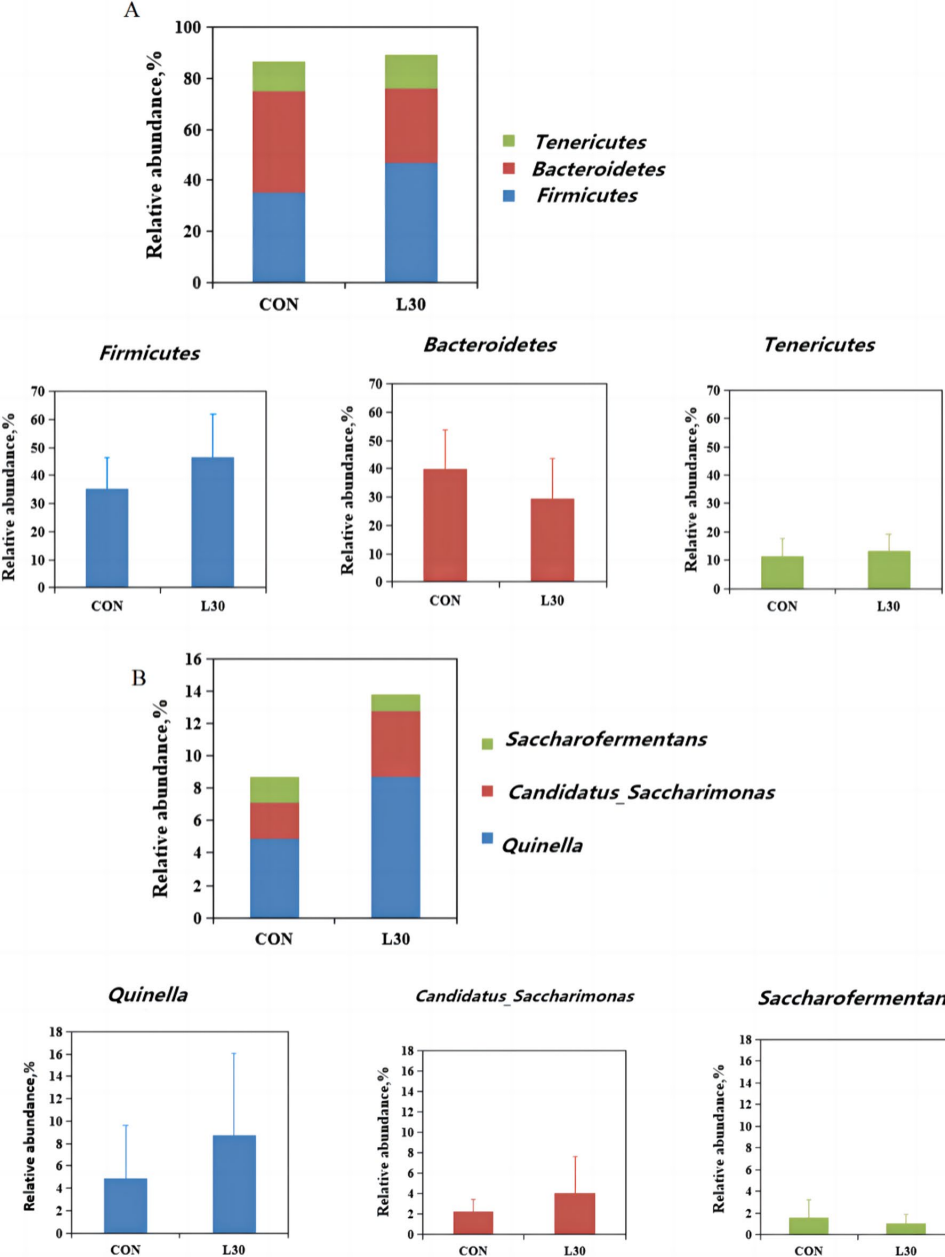
Effect of FHTR on rumen microbiota composition. The rumen micobiota composition of goats in CON and L30 group at phylum (**A**) and genus level (**B**)

In this experiment, the bacteria with more than 0.1% relative abundance in at least one sample were analyzed. The relative abundance of 13 phyla is shown in Table 4. The dominant bacteria were *Firmicutes* (35.12% and 46.52% on average), *Bacteroidetes* (39.80% and 29.47% on average), and *Tenericutes* (11.41% and 13.16% on average) in CON and L30, respectively (Fig. 3A). The relative abundance of 17 general is shown in Table 5. The dominant bacteria were *Quinella* (4.90% and 8.70% on average), *Candidatus_Saccharimonas* (2.25% and 4.11% on average), and *Saccharofermentans* (1.57% and 1.04% on average) in CON and L30, respectively (Fig. 3B). In general, the relative abundance of these bacteria were not affected by diet treatments.

**Table 4:** Effects of FHTR on rumen bacteria (phylum-level) of Chuanzhong black goats (relative abundance ≥ 0.1%)

Items	Treatment		SEM	*P*-value
	CON	L30		
*Firmicutes*	35.12	46.52	14.54	0.233
*Bacteroidetes*	39.80	29.47	3.86	0.190
*Tenericutes*	11.41	13.16	1.62	0.605
*unidentified_Bacteria*	2.53	4.49	0.70	0.259
*Proteobacteria*	2.20	1.69	0.48	0.617
*Lentisphaerae*	1.98	0.61	0.46	0.139
*Melainabacteria*	1.94	0.93	0.30	0.091
*Kiritimatiellaeota*	1.40	0.72	0.29	0.255
*Synergistetes*	0.64	1.06	0.23	0.383
*Planctomycetes*	0.46	0.33	0.12	0.595
*Spirochaetes*	0.68	0.35	0.11	0.152
*Gracilibacteria*	0.45	0.21	0.08	0.144
*Actinobacteria*	0.05	0.21	0.05	0.162

*CON* 0% fermented herbal tea residue silage, *L30* 30% fermented herbal tea residue silage, *SEM* standard error of means

**Table 5:** Effects of FHTR on rumen bacteria (genus-level) of Chuanzhong black goats (relative abundance ≥ 0.1%)

Items	Treatment		SEM	*P*-value
	CON	L30		
*Quinella*	4.90	8.70	1.68	0.272
*Candidatus_Saccharimonas*	2.25	4.11	0.72	0.207
*Saccharofermentans*	1.57	1.04	0.35	0.466
*Succiniclasticum*	1.28	0.23	0.30	0.083
*Methylobacterium*	0.08	0.49	0.24	0.430
*Fretibacterium*	0.6	1.04	0.23	0.363
*unidentified_Bacteroidales*	1.33	1.09	0.21	0.581
*unidentified_Ruminococcaceae*	0.90	0.86	0.13	0.892
*Anaeroplasma*	0.96	0.38	0.17	0.083
*Succinivibrio*	0.42	0.09	0.12	0.169
*unidentified_Lachnospiraceae*	0.92	0.67	0.13	0.345
*unidentified_Prevotellaceae*	0.55	0.43	0.10	0.548
*Cupriavidus*	0.33	0.32	0.11	0.974
*Sphaerochaeta*	0.22	0.13	0.07	0.503
*unidentified_Gracilibacteria*	0.45	0.21	0.08	0.144
*Anaerovorax*	0.39	0.24	0.05	0.167
*Pseudobutyrivibrio*	0.18	0.32	0.06	0.239

*CON* 0% fermented herbal tea residue silage, *L30* 30% fermented herbal tea residue silage, *SEM* standard error of means

### Function prediction of rumen flora with different diets

**Fig. 4:**
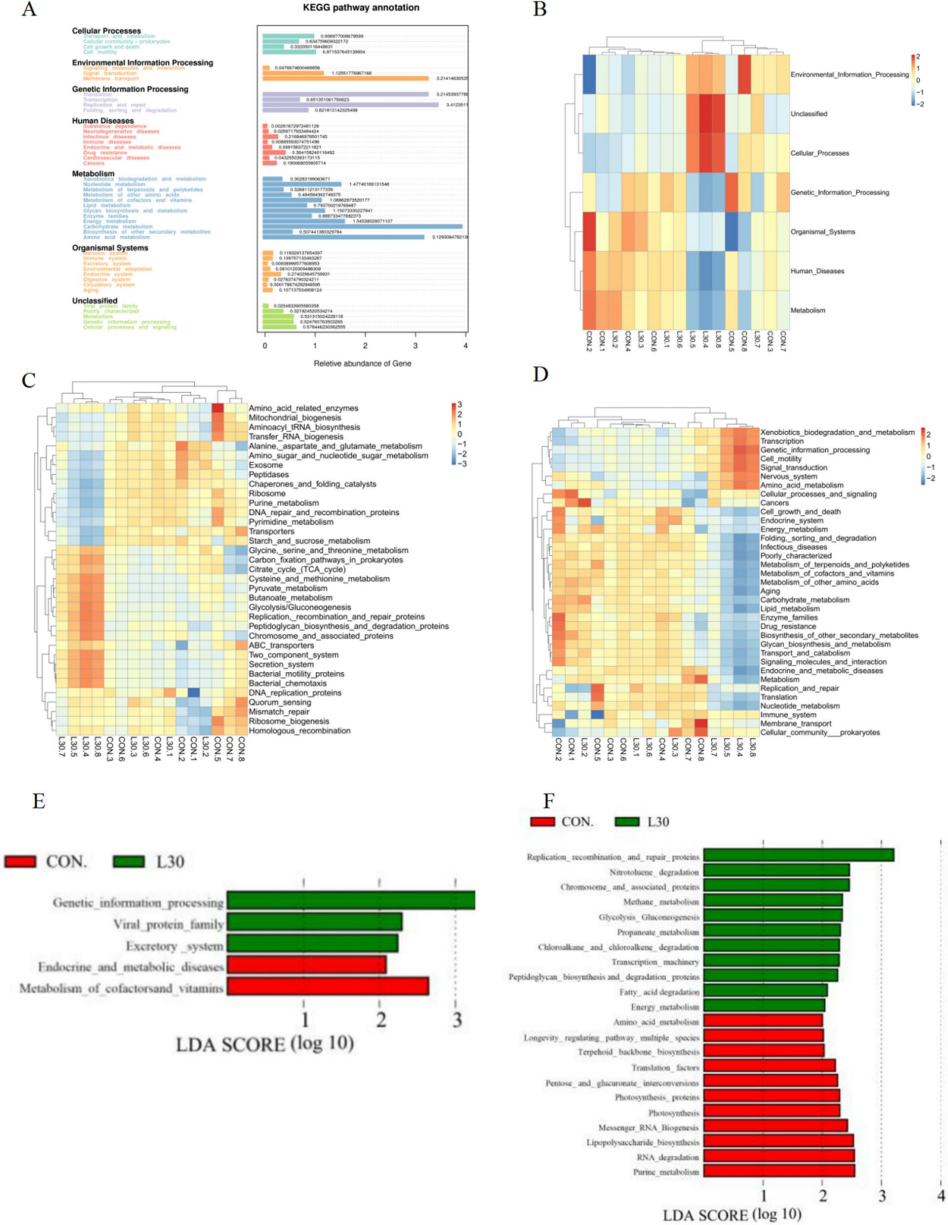
Statistical graph of gene prediction function results of rumen microbiota via Tax4fun at **A**. Cluster heatmap of the relative abundance of rumen microbiota function analysis via Tax4fun at Level 1 (**B**), Level 2 (**C**) and Level 3 (**D**). The LDA Effect Size analysis (LEfSe) (**E** and **F**) of goats in CON and L30 group at Level 2 and Level 3, respectively

The KEGG pathways can be divided into levels 1, 2, and 3. Tax4fun was used to predict the rumen microbial function of different treatment groups (Fig. 4A), and the top 10 pathways at these 3 levels are shown in Fig. 4B–D. In level 1, the top three predicted functions were Metabolism, Genetic information processing, and Environmental information processing; In level 2, the top three predicted functions were Carbohydrate metabolism, Replication and repair, and Translation; in level 3, the top three predicted functions were Transporters, DNA repair and recombination proteins and Two component system. In general, dietary treatments did not alter the priority of these pathways. LDA Effect Size analysis (LEfSe) is an analytical tool used to for discover and interpret high-dimensional data for finding biometric identifiers with statistical differences between groups. As shown in Fig. 3E, F, LEfSe analysis revealed that 4 and 22 pathways are significantly different in level 2 and level 3 from the CON and L30, respectively, among which CON and L30 had 13 pathways each. In level 2, FHTR had significantly higher function enrichment of genetic information processing and excretory system than CON. However, L30 dramatically reduced the function enrichment of metabolism of co-factors and vitamins compared to that in CON. In level 3, L30 had higher function enrichment of replication recombination and repair proteins, methane metabolism, glycolysis and gluconeogenesis, protein degradation, fatty acid degradation, and energy metabolism than CON. Interestingly, dietary FHTR supplementation reduced the function enrichment of amino acid metabolism and longevity regulating pathway.

## Discussion

### Carcass characteristics and meat quality

Meat quality is an important economic trait of goats husbandry. Crude protein and moisture content are the most important characteristics for evaluating meat production (Tang et al. 2021). In the present study, the inclusion of FHTR in the diet had significant effect on muscle moisture content and crude protein, indicated that the addition of FHTR could improve the meat quality of goats. Moreover, meat color is the most important quality attribute that influences the purchasing decisions of consumers (Carpenter et al. 2001). Although the color of muscles has little correlation with meat flavor, consumers usually prefer fresh meat with bright red color (Zhang et al. 2015). In the present work, the color index a* of meat was higher in FHTR than in CON. As previously reported, the value of a* is affected by the ratio of deoxymyoglobin to oxymyoglobin (Campos et al. 2017). Myoglobin is a small molecule pigment protein that can reversibly bind to oxygen. Moreover, the color values of L* and b* had no significant effect in the two treatment groups, which contradicted the findings of a previous study (Li et al. 2018). FHTR contains a number of antioxidant active substances, such as rosmarinic acid and chlorogenic acid (Wang et al. 2021b). These acids have significant application potential in meat products because they can reduce, the malondialdehyde content and improve the meat quality (Li et al. 2019). Other factors can affect meat quality, such as natural dietary antioxidants have been used to improve meat quality by improving the antioxidant status via enhanced scavenging capacity and reduced capacity of DPPH and ABTS free radicals (Salami et al. 2016). In the present study, the FHTR group had a lower water loss rate than CON. When exposed to air, the disulfide bond cross-linking between the heavy chains of myofibril in meat resulted in a significant reduction in meat tenderness and water retention (Wang et al. 2020). In conclusion, the changes in the meat quality of goats could be due to the large amounts of active substances in FHTR. However, the molecular mechanism of FHTR in affecting meat quality needs further investigation.

### Serum biochemical indices

Serum biochemical indicators indicate the utilization of nutrients in the body. ALT and LDH are used to assess whether the liver’s ability to metabolize proteins and amino acids is abnormal (Jiang et al. 2014). In the present work, the serum ALT and LDH levels were not different between the groups (P > 0.05), indicating that the addition of FHTR did not damage the liver function of Chuanzhong black goats. CR is a metabolite of creatine and phosphocreatine, and its level can reflect the absolute amount of skeletal muscle. In general, CR can be used as a marker of nutrition and muscle quality (Yoshida et al. 2019). In current study, on day 35, goats fed diets with 30% FHTR had a greater (P < 0.05) CR concentration, indicating that adding FHTR can improve muscle quality.

### Rumen fermentation

Rumen pH and VFA content are essential index for assessing ruminant fermentation (Cui et al. 2022). In this study, FHTR substituting for WCS significantly decreased the ruminal pH, which may be related to changes in rumen VFA. VFA plays a role in maintaining and providing energy for the growth of ruminants. The structure, quality, microbial activity, and microflora of ruminant diets affect the composition and concentration of VFA in the rumen (Sun et al. 2019). The digestion, absorption, and utilization of nutrients by ruminants can change (Lee 1985). AA is a product of fiber degradation and a main carbon resource for the synthesis of milk fat and body fat (El-Essawy et al. 2019). PA, as a product of starch fermentation, is the precursor of glucose synthesis in ruminants(Maldini et al. 2019). The addition of FHTR led to increases in the concentration of AA and the ratio of AA to PA. Krause et al. found that increasing the dietary particle size decreased the total rumen VFA in mid-lactating dairy cows and increased the ratio of AA to PA (Krause et al. 2002). The ratio of AA to PA may indicate an increase in energy efficiency in organisms (Shah et al. 2019). At present, few studies have been conducted to determine the regulatory mechanism of FHTR in the rumen. FHTR may improve rumen fermentation by considerable proportion of the nutrients and bioactive compounds mechanism. Based on the current results, the supplementation of FHTR in the diet promoted rumen fermentation, energy conversion, and absorption, resulting in a positive effect on the body.

### Rumen microbiota

The rumen microbiota is closely concerned to the host’s overall metabolism and immune system (Jami et al. 2013). In the present study, no conspicuous differences were found in the richness indices (Chao1 and Ace), PD whole tree, observed species, and diversity indices (Shannon and Simpson), indicating the FHTR had no significant effect on the alpha diversity of the rumen microbiota. Nevertheless, by combining the PCA and UPGMA clustering results, we found that FHTR significantly changed the beta diversity of the rumen microbiota,which by contrast Zhuang et al. (2021) reported that feeding fattening cattle with FHTR, did not significantly affect the beta diversity of the fecal microbiota. These results indicated that the addition of FHTR had a certain effect on the diversity of rumen and fecal microorganisms. Different diets did not alter the fact that *Firmicutes* and *Bacteroidetes* are the most abundant bacteria in the goat rumen (Wang et al. 2021b). In the present study, *Firmicutes*, *Tenericutes*, and *Bacteroidetes* were the three dominant phyla, accounting for more than 80% of the microorganisms in the rumen. They are the most important phyla, consistent with previous findings (Sadet-Bourgeteau et al. 2010). *Bacteroidetes* are the main flora that assist organisms to digest carbohydrates. *Firmicutes* are the principal cellulose decomposers (Zhang et al. 2015). No significant differences were found in the relative abundance of *Bacteroidetes* and *Firmicutes* between the two groups. However, *Firmicutes* increased and *Bacteroidetes* decreased in the FHTR group compared with those in the CON group. Some researchers reported that the increase in *Firmicutes* and the decrease in *Bacteroidetes* can lead to obesity in mice (Stojanov et al. 2020). Hence, dietary supplementation with FHTR may promote the growth of goat. At the genus level, the rumen bacteria with relative abundance greater than 0.1% were selected for analysis. These genera were not affected by the diet. In present study, we found that *Quinella* was the primary bacteria in the rumen. Han et al. (2022) found that *Rikenellaceae_RC9*_*gut_* group and *Quinella* were correlated with different altitudes and rumen fermentation parameters, suggesting that they may play a key role in the adaptation to extreme environments. Hence, *Quinella* are beneficial bacteria that regulate rumen fermentation. In general, FHTR can improve the structure and diversity of rumen bacteria. The relative abundance of *Firmicutes*, *Bacteroidetes*, and *Quinella* may serve as a biomarker of the rumen microbial structure, and the microbial structure of goats fed with FHTR becomes complete.

As expected, differences in the rumen microbiota significantly affected the microbial function. Tax4fun is one of the most essential microbial community functions. The 16S high-throughput sequencing data were available for classifying OTU species in the SILVA database. Based on the classification results, the 16S copy number was standardized according to the function of the microbial community was predicted by constructing the linear relationship between the SILVA classification and the prokaryotic classification in the KEGG database realized. With the changes in the rumen microflora, LEfSe analysis showed that several pathways were enriched in goats fed with FHTR; these pathways include methane metabolism, glycolysis and gluconeogenesis, protein degradation, fatty acid degradation, and energy metabolism, consistent with the findings of Li et al. (2021b). It is worth emphasizing that the relative abundance of glycolysis, gluconeogenesis, and energy metabolism pathways in the FHTR group were significantly increased. Pan et al. (2021) found that nutrient-related metabolism was enriched in the second rumen development, whereas differentially expressed genes of the rumen microbiome were enriched in glycolysis/gluconeogenesis activities. This phenomenon may lead to higher concentrations of BA and AA in the rumen. Research on other differential KEGG orthologs remains limited. The regulation of rumen fermentation and the underlying mechanism need further investigation.

In conclusion, FHTR supplementation in diet could reduce the muscle shear force and water loss rate and improve rumen fermentation. We speculated that FHTR can can affect the meat quality, serum indices, improve rumen fermentation by regulating the rumen microbial function and increasing the abundance of glycolysis and gluconeogenesis, protein degradation, fatty acid degradation, and energy metabolism pathways. This finding might be related to a variety of biologically active substances in FHTR. However, the specific mechanism needs further study.

### Supplementary Information

**Additional file 1: Table S1.** Nutritional composition of the fermented herbal tea residue (FHTR) (Dry matter basis). **TableS2.** Composition and nutrient levels of the experimental diets (Dry matter basis). **Table S3.** Effects of FHTR on amino acid score (AAS) of LD of Chuanzhong black goats. **Table S4.** Effects of FHTR on serum indices of Chuanzhong black goats. **Table S5.** Differential Analysis of Rumen Microorganisms.

## Data Availability

The raw sequencing data from this study have been deposited in the Genome Sequence Archive in Beijing Institute of Genomics (BIG) Data Center (https://bigd.big.ac.cn/), under the accession number: CRA005725.

## Abbreviations

HTR: Herbal tea residue
FHTR: Fermented herbal tea residue
DM: Dry matter
CP: Crude protein
EE: Ether extract
pH: pH value
VFA: Volatile fatty acids
NH_3_–N: Ammonia nitrogen
AA: Acetic acid
PA: Propionic acid
IBA: Isobutyric acid
BA: Butyric acid
VA: Valeric acid
EAA: Essential amino acid
NEAA: Nonessential amino acid
DAA: Delicious amino acid
TAA: Total amino acids
Thr: Threonine
Val: Valine
Met: Methionine
Ile: Isoleucine
Leu: Leucine
Phe: Phenylalanine
Lys: Lysine
Asp: Aspartic acid
Ser: Serine
Glu: Glutamic acid
Gly: Glycine
Ala: Alanine
Cys: Cystine
Tyr: Tyrosine
His: Histidine
Arg: Arginine
Pro: Proline
L∗: Lightness
a∗: Redness
b∗: Yellowness
ALT: Aminotransferase
UA: Uric acid
CR: Creatinine
GLU: Glucose
CK: Creatine kinase
LDH: Lactate dehydrogenase
LD: Longissimus dorsi
