# Supplementary material for: Effects of fermented herbal tea residue on meat quality, rumen fermentation parameters and microbes of black goats
Source: AMB Express. 2023 Oct 3;13:106. doi: 10.1186/s13568-023-01610-2 (PMC10547668; doi:10.1186/s13568-023-01610-2)
Supplement: Supplementary file 1 — Additional file 1: Table S1. Nutritional composition of the fermented herbal tea residue (FHTR) (Dry matter basis). TableS2. Composition and nutrient levels of the experimental diets (Dry matter basis). Table S3. Effects of FHTR on amino acid score (AAS) of LD of Chuanzhong black goats. Table S4. Effects of FHTR on serum indices of Chuanzhong black goats. Table S5. Differential Analysis of Rumen Microorganisms. [file 13568_2023_1610_MOESM1_ESM.docx]

**Table S1** Nutritional composition of the fermented herbal tea residue (FHTR) (Dry matter basis)

| Items | Content |
| --- | --- |
| Nutritional ingredient% |  |
| DM | 26.58 |
| CP | 8.85 |
| EE | 2.82 |
| Ash | 12.17 |
| NDF | 54.29 |
| ADF | 34.33 |

DM, Dry matter; CP, Crude protein; EE, Ether extract; NDF, Neutral detergent fiber; ADF, Acid detergent fiber.

**Table S2** Composition and nutrient levels of the experimental diets (Dry matter basis)

| Items | Dietary treatment | |
| --- | --- | --- |
|  | CON | L30 |
| Ingredient |  |  |
| Whole plant corn silage | 76.20 | 53.30 |
| Fermented herbal tea residue | 0.00 | 22.90 |
| Peanut seedling feed | 19.00 | 19.00 |
| Soya bean meal | 1.20 | 1.20 |
| Alfalfa | 0.47 | 0.47 |
| Mountain flour | 0.10 | 0.10 |
| Salt | 0.05 | 0.05 |
| Nutritional ingredient% | | |
| DM | 30.84 | 31.33 |
| CP | 11.68 | 11.79 |
| EE | 0.01 | 0.02 |
| Ash | 0.13 | 0.12 |
| NDF | 69.23 | 68.76 |
| ADF | 52.21 | 51.49 |
| Ca | 0.90 | 1.04 |
| P | 0.44 | 0.41 |
| NEM mac/kg | 1.18 | 1.18 |
| NEG mac/kg | 0.62 | 0.62 |

CON, 0% fermented herbal tea residue silage; L30, 30% fermented herbal tea residue silage; DM, Dry matter; CP, Crude protein; EE, Ether extract; NDF, Neutral detergent fiber; ADF, Acid detergent fiber; Ca, Calcium; P, Phosphorus. NEM, Net energy for maintenance; NEG, Net energy for weight gain.

**Table S3** Effects of FHTR on amino acid score (AAS) of LD of Chuanzhong black goats

| Items | WHO/FAO | Treatment | | | |
| --- | --- | --- | --- | --- | --- |
|  |  | CON | | L30 | |
|  |  | Content | AAS, (%) | Content | AAS, (%) |
| Lys | 5.50 | 7.03 | 127.8 | 7.15 | 130.0 |
| Leu | 7.00 | 6.43 | 91.9 | 6.52 | 93.1 |
| Ile | 4.00 | 3.59 | 89.8 | 3.65 | 91.3 |
| Thr | 4.00 | 3.55 | 88.8 | 3.59 | 89.8 |
| pHe +Tyr | 6.00 | 6.78 | 113.0 | 6.93 | 115.5 |
| Met +Cys | 3.50 | 2.79 | 79.7 | 2.90 | 82.9 |

CON, 0% fermented herbal tea residue silage; L30, 30% fermented herbal tea residue silage; Lys, Lysine; Leu, Leucine; Ile, Isoleucine; Thr, Threonine; Phe, Phenylalanine; Met, Methionine; Cys, Cystine; WHO, World Health Organization; FAO, Food and Agriculture Organization of the United Nations; AAS, amino acid score.

**Table S4** Effects of FHTR on serum indices of Chuanzhong black goats

| Items | Treatment | | SEM | *P-value^1^* | *P-value^2^* |
| --- | --- | --- | --- | --- | --- |
|  | CON | L30 |  |  |  |
| UA, (μmol/L) |  |  |  |  |  |
| 0d | 7.20 | 7.60 | 0.22 | 0.934 | 0.486 |
| 17d | 6.80 | 7.40 |  |  | 0.583 |
| 35d | 7.80 | 8.00 |  |  | 0.740 |
| LDH, (U/L) |  |  |  |  |  |
| 0d | 401.00 | 385.80 | 17.29 | 0.233 | 0.729 |
| 17d | 406.40 | 340.20 |  |  | 0.165 |
| 35d | 329.60 | 410.20 |  |  | 0.367 |
| GLU, (mmol/L) |  |  |  |  |  |
| 0d | 3.16 | 3.32 | 0.14 | 0.550 | 0.724 |
| 17d | 1.88 | 1.47 |  |  | 0.144 |
| 35d | 2.25 | 2.58 |  |  | 0.680 |
| CR, (μmol/L) |  |  |  |  |  |
| 0d | 41.00 | 38.20 | 1.71 | 0.119 | 0.524 |
| 17d | 48.80 | 50.60 |  |  | 0.804 |
| 35d | 40.80^a^ | 55.40**^b^** |  |  | 0.045 |
| CK, (U/L) |  |  |  |  |  |
| 0d | 321.66 | 282.08 | 11.59 | 0.796 | 0.508 |
| 17d | 284.02 | 282.68 |  |  | 0.966 |
| 35d | 255.62 | 239.64 |  |  | 0.551 |
| ALT, (U/L) |  |  |  |  |  |
| 0d | 15.02 | 13.30 | 1.02 | 0.301 | 0.483 |
| 17d | 16.06 | 15.58 |  |  | 0.856 |
| 35d | 14.82 | 20.50 |  |  | 0.291 |

CON, 0% fermented herbal tea residue silage; L30, 30% fermented herbal tea residue silage; UA, Uric acid; LDH, Lactate dehydrogenase; GLU, Glucose; CR, Creatinine; CK, Creatine kinase; ALT, Alanine aminotransferase; SEM, Standard error of means. P-value1 represent p-values for interaction of feeding time and diet. P-value2 represents the p value of the T-test of the diet.

**Table S5** Differential Analysis of Rumen Microorganisms

| Group | MRPP | | ANOSIM | | PERMANOVA | |
| --- | --- | --- | --- | --- | --- | --- |
| CON vs L30 | A | *P-value* | *R* | *P-value* | *R^2^* | *P-value* |
|  | 0.05542 | 0.001 | 0.4062 | 0.001 | 0.1604 | 0.002 |

CON, 0% fermented herbal tea residue silage; FHTR, 30% fermented herbal tea residue silage.
